# Upstream Open Reading Frame Mediated Translation of WNK8 Is Required for ABA Response in *Arabidopsis*

**DOI:** 10.3390/ijms221910683

**Published:** 2021-10-01

**Authors:** Zhiyong Li, Yajuan Fu, Jinyu Shen, Jiansheng Liang

**Affiliations:** 1 Department of Biology, Southern University of Science and Technology, Shenzhen 518055, China; 11930723@mail.sustech.edu.cn (Y.F.); shenjy@mail.sustech.edu.cn (J.S.); 2 Key Laboratory of Molecular Design for Plant Cell Factory of Guangdong Higher Education Institutes, Department of Biology, Southern University of Science and Technology, Shenzhen 518055, China; 3 Academy for Advanced Interdisciplinary Studies, Southern University of Science and Technology, Shenzhen 518055, China

**Keywords:** upstream open reading frame, translation, abscisic acid, protein kinase WNK8

## Abstract

With no lysine (K) (WNK) kinases comprise a family of serine/threonine kinases belonging to an evolutionary branch of the eukaryotic kinome. These special kinases contain a unique active site and are found in a wide range of eukaryotes. The model plant *Arabidopsis* has been reported to have 11 WNK members, of which WNK8 functions as a negative regulator of abscisic acid (ABA) signaling. Here, we found that the expression of WNK8 is post-transcriptionally regulated through an upstream open reading frame (uORF) found in its 5′ untranslated region (5′-UTR). This uORF has been predicted to encode a conserved peptide named CPuORF58 in both monocotyledons and dicotyledons. The analysis of the published ribosome footprinting studies and the study of the frameshift CPuORF58 peptide with altered repression capability suggested that this uORF causes ribosome stalling. Plants transformed with the native *WNK8* promoter driving WNK8 expression were comparable with wild-type plants, whereas the plants transformed with a similar construct with mutated CPuORF58 start codon were less sensitive to ABA. In addition, WNK8 and its downstream target RACK1 were found to synergistically coordinate ABA signaling rather than antagonistically modulating glucose response and flowering in plants. Collectively, these results suggest that the WNK8 expression must be tightly regulated to fulfill the demands of ABA response in plants.

## 1. Introduction

Protein kinases comprise a superfamily of enzymes that regulate a wide range of cellular processes through induced phosphorylation of downstream protein substrates in all living cells. WITH NO LYSINE (WNK) kinases belong to a superfamily of serine/threonine protein kinases uniquely characterized by the lack of a catalytic lysine residue in the kinase subdomain II, which is crucial for coordinating ATP and catalyzing phosphoryl transfers within other protein kinase superfamily and downstream targets [1].

WNK kinases have been identified in various eukaryotes. They were first discovered in mammals while screening for new members of the mitogen-activated protein, serine/threonine kinase (MAPK) family. Humans have four different WNK kinases, namely WNK1, WNK2, WNK3, and WNK4 [2]. Studies have reported four WNK members in mice, two in *Xenopus*, and a single WNK homolog member in *Drosophila melanogaster*, *Caenorhabditis elegans*, and *Giardia lambia* [3,4,5]. In animals, WNK kinases play critical roles in ion homeostasis by regulating numerous ion channels, including sodium chloride cotransporter (NCC) and sodium potassium chloride cotransporter (NKCC1), as well as in angiogenesis, tumor cell growth, and neuropathic disorders [6,7,8,9,10,11,12]. In plants, the WNK gene family is larger and more diverse than in animals. A total of 11 and 9 WNK members have been identified in the model plant *Arabidopsis* and *Oryza sativa*, respectively [13,14]. An even larger number of WNK members have been found in peach (18) and soybean (26) [15,16]. Plant WNK family members have been implicated to play important roles in many plant physiological and developmental processes, including the regulation of flowering time [13,17], fruit development and ripening [16], internal circadian rhythms [18,19,20], root system architecture [15], cellular pH homeostasis, [21] and various abiotic and biotic stress [22,23,24,25,26,27].

The plant hormone abscisic acid (ABA) is known to play important roles in development and stress responses. Some WNK family members have been found to be involved in ABA-signaling pathway. WNK8 and WNK9 from *Arabidopsis* have been shown to regulate salt and osmotic stress responses through an ABA-dependent pathway [23,24]. Studies have shown that *AtWNK8* expression is enhanced under salt and sorbitol treatments and that the *wnk8* mutant lines have higher proline content and exhibit significant catalase (CAT) and peroxidase (POD) activities, which lead to in high salinity and osmotic stress tolerance [23]. Furthermore, *AtWNK8* has been proposed to negatively modulate ABA signaling by interacting with ABA-signaling core components, including the ABA receptor, PYR1, and type 2C protein phosphatase (PP2CA) [28]. In contrast, *AtWNK9* positively regulates ABA signaling and enhances drought tolerance in transgenic plants [24]. *OsWNK9*, a member of rice WNK gene family, has been functionally well characterized, and its overexpression in *Arabidopsis* has been shown to confer high tolerance to drought and salt stress in an ABA-dependent manner [25]. Moreover, a previous study showed that the soybean *GmWNK1* gene reduces sensitivity to both ABA and mannitol treatment with increased endogenous ABA in *Arabidopsis* [15]. Furthermore, this study showed that GmWNK1 directly interacted with a crucial ABA catabolism enzyme, GmCYP707A1. Collectively, these findings revealed the diverse roles of plant WNKs in ABA response and the relevance of their interaction with ABA in both signaling pathways and ABA metabolic processes. However, many questions regarding the regulation of WNK kinases and their comprehensive interaction with ABA components remain to be explored.

In the present study, we investigated *Arabidopsis* WNK8 found that two individual T-DNA insertion lines of *WNK8* showed similar ABA response phenotypes. However, no significant transcript regulation of *WNK8* was observed under ABA treatment compared to the control. Further studies showed that a conserved peptide encoded by an upstream open reading frame (uORF), called uORF58, has been identified in the 5′-UTR of *WNK8*. We showed that the translation of WNK8 was repressed by CPuORF58 in vivo and that this element may act by a ribosome stalling mechanism, independently of the main open reading frame (mORF) downstream of the uORF. Moreover, we showed that such an ingenious regulation is necessary for plants to fulfill the demands of ABA response caused by the uncontrolled expression of WNK8. Finally, we found that WNK8 coordinates ABA signaling with RACK1, a downstream target of WNK8.

## 2. Results

### 2.1. WNK8 Negatively Regulates ABA Response in Seed Germination and Post-germination Development

**Figure 1:**
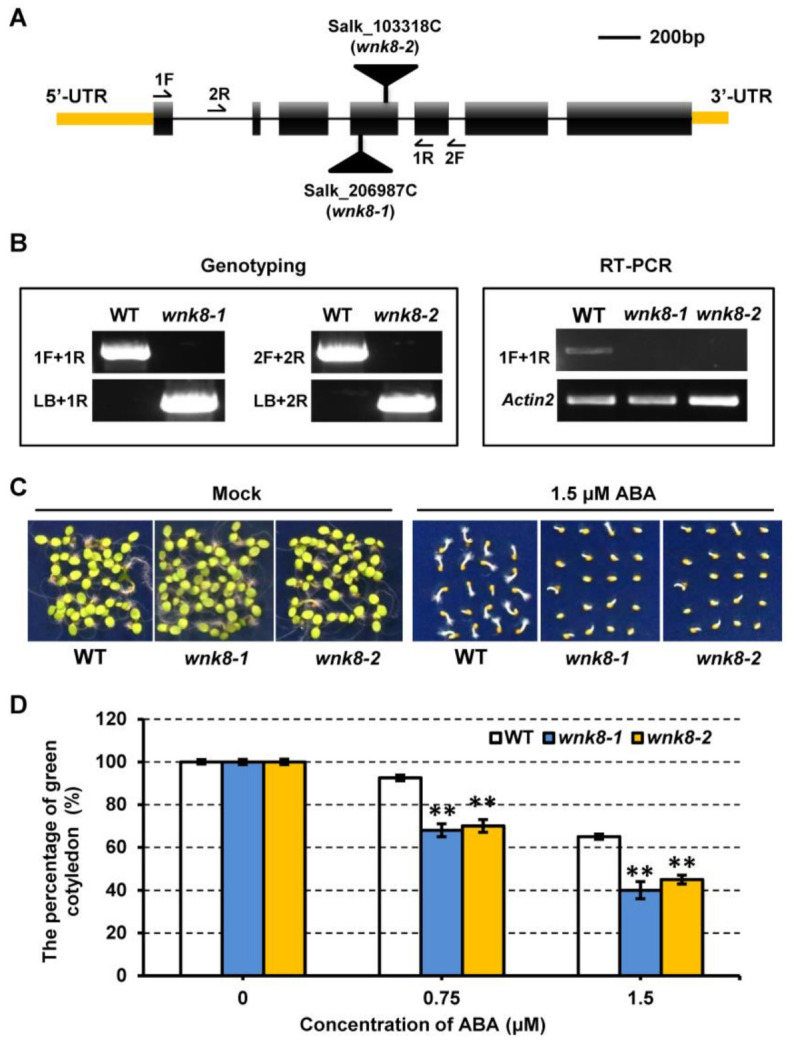
WNK8 negatively regulates ABA response during seed germination and post-germination development. (**A**) Schematic presentation of the *WNK8* gene. 5′ and 3′-UTR are highlighted with yellow boxes. Introns are depicted by solid lines and exons are indicated by black boxes. Two black triangles represented the T-DNA insertion sites of respective *wnk8* alleles. Positions of oligonucleotides used for genotyping and RT-PCRs in (**B**) are indicated by arrows. (**B**) Genotyping and RT-PCRs analysis in Col-0 wild-type (WT) and *wnk8* alleles. *Actin2* was used as the internal control. (**C**) Representative images for seed germination of Col-0 and *wnk8* alleles in the absence or presence of ABA. (**D**) Cotyledon greening rates of indicated genotypes when grown on media supplemented with different concentration of ABA. Data indicate repeat experiments (*n* = 4). Asterisks indicate significant differences to WT.

A previous study showed that different T-DNA insertion mutants of *WNK8* led to opposite ABA responses during seed germination [28]. To learn more about the roles of WNK8 in response to ABA, two T-DNA insertion mutants, SALK_206987C (*wnk8-1*) and SALK_103318C (*wnk8-2*), were used in this study (Figure 1A). The T-DNA insertions were confirmed to be present in the fourth exon of the *WNK8* gene by genotyping and sequencing (Figure 1B). An RT-PCR analysis using region-specific primers detected no *WNK8* transcript in either allele (Figure 1B).

We further carried out a seed germination assay for each genotype following ABA treatment. The germination rates of the different genotypes were similar in the absence of ABA. In the presence of 1.5 μM ABA, both *wnk8-1* and *wnk8-2* seeds showed higher ABA sensitivity with much lower germination rates than the wild-type seeds (Figure 1C). In addition, the *wnk8-1* and *wnk8-2* mutants showed significantly lower cotyledon greening rates after germination in the medium with ABA for 7 days (Figure 1D). Taken together, these results suggest that WNK8 negatively regulates the ABA response during seed germination and post-germination development.

### 2.2. WNK8 Has a Conserved Open Reading Frame in Its 5′-UTR

To study the expression pattern of *WNK8* in the presence of ABA, we quantified *WNK8* mRNA accumulation under high ABA concentration (50 μM) and found only slightly increase of*WNK8* mRNA levels (Figure S1). This indicated that the regulatory mechanism of *WNK8* at high ABA concentrations is unlikely to be involved in transcription.

To learn more about the expression of WNK8, transcription and translation were further investigated using the ATHENA database (http://athena.proteomics.wzw.tum.de accessed on 16 August 2021), a collection of many protein and transcript expression profiles from *Arabidopsis thaliana* (Col-0) plants [29]. The ATHENA search analysis showed that similar transcription levels of *WNK8* in different organs or tissues, except for higher transcriptional expression in pollen (Figure S2A). Consistent with the data from ATHENA, the qRT-PCR results confirmed higher expression levels of *WNK8* in open flowers and mature pollens (Figure S2B). In contrast, the translation levels of WNK8 varied greatly among the organs or tissues, in which it was almost undetectable, such as in cotyledons (CT), hypocotyl (HY), rosette leaf (LFs), and carpel (CP). This suggests a post-transcriptional regulation mechanism for *WNK8*.

**Figure 2:**
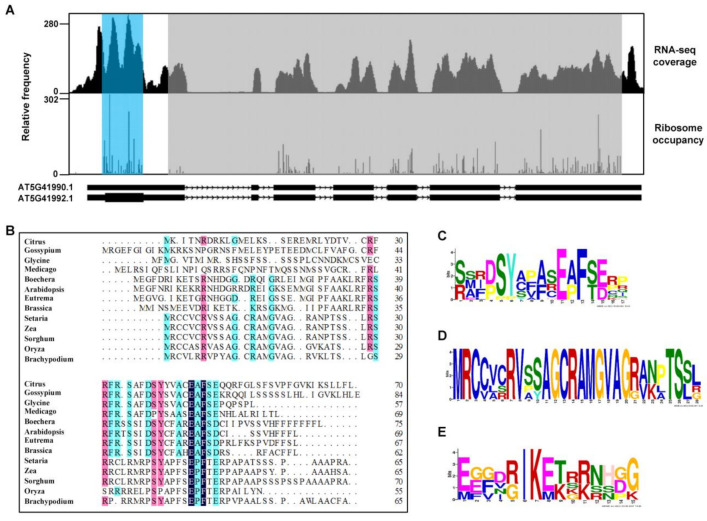
Conserved open reading frame is found in the 5′-UTR of WNK8. (**A**) Comparative ribosome footprinting profile of *WNK8*. The coverage of RNA-seq reads is shown in upper panel and the ribosome occupancy is shown in lower panel. Light blue: CPuORF58; Gray: main ORF; White: UTR. (**B**) Sequence alignment of the predicted amino acid sequences of CPuORF58 from different plant species. The sequence logos are resulted from the sequence alignment of the uORFs from all selected plant species (**C**), monocts (**D**), and dicots (**E**). Letter height indicates the frequency in the alignment.

The presence of an encoded peptide in the 5′-UTR of *WNK8* upstream of its mORF has been previously reported [30]. We further analyzed the published ribosome profiling data for the distribution of ribosomes along the *WNK8* sequence [31,32,33]. The data clearly indicated that the uORF had a high frequency of ribosome occupancy, with an average higher ribosome density compared to that of the mORF (Figure 2A). We further performed an in silico analysis of WNK8 homologs within representative plant species to determine the functionality of such a peptide. The basic local alignment search tool BLASTP retrieved 12 nucleotide sequences encoding WNK8 homologs from both monocots and dicots, using the WNK8 protein sequence as the query. These homologous sequences were further analyzed for the presence of ORFs, and this analysis led to the identification of 12 uORFs (Figure 2B). Further alignment of these sequences showed a certain degree of conservation in both monocots and dicots (Figure 2C). More peptide conservation was observed in monocots than in dicots (Figure 2D,E and Figure S3).

### 2.3. CPuORF58 Is Essential for the Translational Suppression of the WNK8

**Figure 3:**
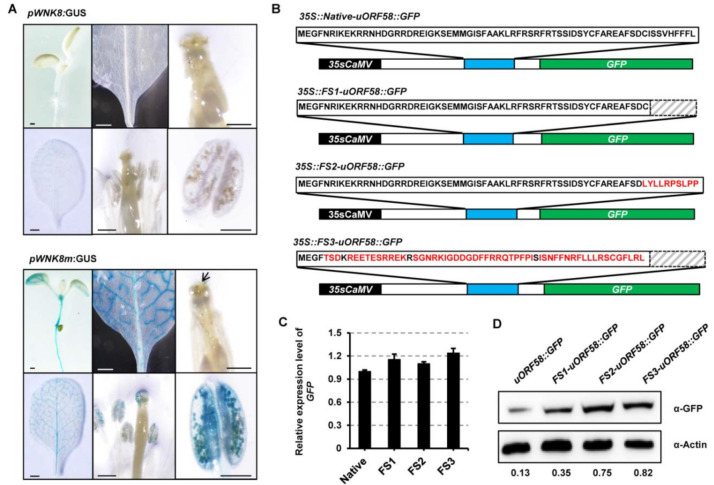
Mutated CPuORF58 enhance the translation of different downstream mORFs. (**A**) GUS expression analyzed by histochemical detection in different stages of seedlings or plants transformed with native promoter *pWNK8:GUS* or site-mutated promoter *pWNK8m:GUS*. (**B**) Schematic representation of the constructs for *Arabidopsis* transformation. The amino acid sequences of the native and mutated uORF with different ones in red were shown at the bottom. FS is short for frame shift; the black box represented 35S CaMV promoter; the white boxes are for *WNK8* 5′-UTR; the light blue boxes are shown as CPuORF58; the green boxes are for GFP ORFs. The expression levels of *GFP* were analyzed using qRT-PCR (**C**) and immunoblotting (**D**) of plants transformed with indicated genetic constructs. Actin2 was used as the internal control.

5′-UTRs play a significant role in translational regulation [33]. We presumed that the CPuORF58 peptide located in the 5′-UTR could play a translational regulatory role in controlling the expression of WNK8. To address this hypothesis, we generated mutations to replace the start codon (ATG) of CPuORF58 with AAG. Two different genetic constructs were generated with the expression of the *GUS* reporter gene controlled by the *WNK8* promoter including its 5′-UTR with the native (*pWNK8:GUS*) or mutated CPuORF58 (*pWNK8m:GUS*). These constructs were transformed into Col-0 *Arabidopsis* plants. Several independent lines were generated for both constructs, and homozygous lines were established. Subsequently, the lines showing similar *GUS* transcript levels were selected (Figure S4) and assayed histochemically for GUS expression in the same tissues (cotyledons, rosette leaves, inflorescence, and anthers). The results showed that weak GUS expression was detected in the plants transformed with *pWNK8*:*GUS*, whereas a strong GUS color was detected in all selected tissues and organs of the plants transformed with *pWNK8m:GUS* (in which CPuORF58 was mutated) (Figure 3A).

The ribosome profiling data presented some peak distributions in the CPuORF58 peptide of WNK8, indicating ribosome stalling (Figure 2A). A previous study showed that the nascent peptides encoded by uORFs cause ribosomal arrest during mRNA translation [34]. Therefore, we decided to examine the importance of the CPuORF58 sequence followed by the generation of several genetic constructs with the truncated or frameshift uORF. A schematic representation of these constructs presented in Figure 3B. In *35S:FS1-uORF58:GFP* and *35S:FS2-uORF58:GFP*, the C-terminus of uORF58 was truncated and shifted, respectively. In *35S:FS3-uORF58:GFP*, the nucleotide sequence of *uORF58* exhibited little change, whereas the deduced amino acid sequence was completely changed. These constructs were transformed into Col-0 *Arabidopsis* plants and homozygous lines were established. Stable transgenic lines with these constructs showing similar *GFP* transcript levels were selected (Figure 3C). The GFP expression was analyzed by ‘immunoblotting. The results showed that both truncated and frameshift mutations in uORF58 led to increased GFP levels, suggesting the involvement of uORF58 in translational suppression of WNK8 (Figure 3D).

### 2.4. Two Independent Regions in the 5′-UTR of WNK8 Are Required for Gene Expression

**Figure 4:**
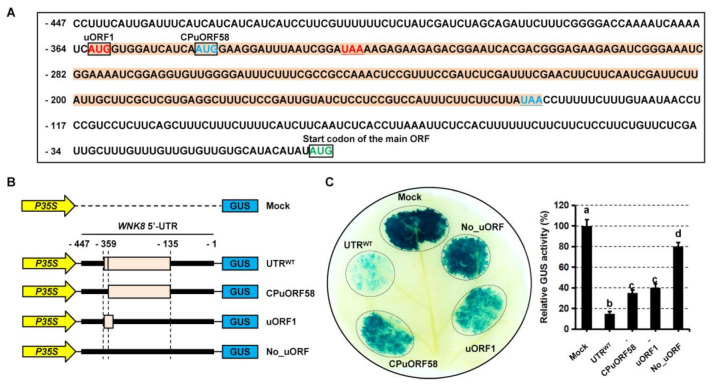
Effects of uORF disruptions in *WNK8* 5′-UTR on downstream gene expression. (**A**) Nucleotide sequence of the *WNK8* 5′-UTR. Sequences in yellow represent uORFs. The start codons for two uORFs and the main ORF are colored and in frame with boxes. Stop codons of uORFs are underlined and highlighted in color same to that of corresponding start codons. (**B**) Schematic representations of the constructs for transient expression. Thick black lines represent the *WNK8* 5′-UTR and pink boxes represents the uORFs, respectively. The constructs at the right represent point mutations (AUG to AAG) in the start codons of *WNK*8 5′-UTR. (**C**) Transient expression analysis in tobacco leaves with the DNA constructs. Illustrative photograph of tobacco leaves transformed with the indicated constructs and visualized with GUS histochemistry. In addition, relative reporter activities are shown at right and different groups marked with letters represent the 0.05 significance level.

A further analysis showed that the 5′-UTR of *WNK8* contains two short open reading frames (here named uORF1 and CPuORF58) (Figure 4A). Regarding the presence of the two uORFs, we constructed a list of site-mutated *WNK8* 5′-UTR to evaluate responsible role of these regions in gene expression regulation (Figure 4B). The GUS expression driven by 35S CaMV promoter was applied as the control. The wild-type (UTR^WT^) and site-mutated of *WNK8* 5′-UTR were inserted between the 35S promoter and the GUS reporter gene. All constructs were introduced into GV3101, and a transient expression assay of GUS expression was performed within tobacco leaves, and followed by GUS histochemistry. The result showed that GUS expression under 35S promoter was strong in control. In contrast, the wild-type of *WNK8* 5′-UTR (UTR^WT^) significantly repressed GUS expression (Figure 4C). The individual site-mutated of *WNK8* 5′-UTR was also able to repress GUS expression driven but less effect in compared with the wild-type 5′-UTR, similar to the inhibition seen from the CPuORF58 in transgenic *Arabidopsis* plants. Unexpectedly, the extent of repression in GUS expression was not enhanced in the double site-mutated UTR^m1+m2^ of *WNK8* 5′-UTR.

### 2.5. The Absence of a Strictly Regulated Expression of WNK8 Altered ABA Sensitivity

**Figure 5:**
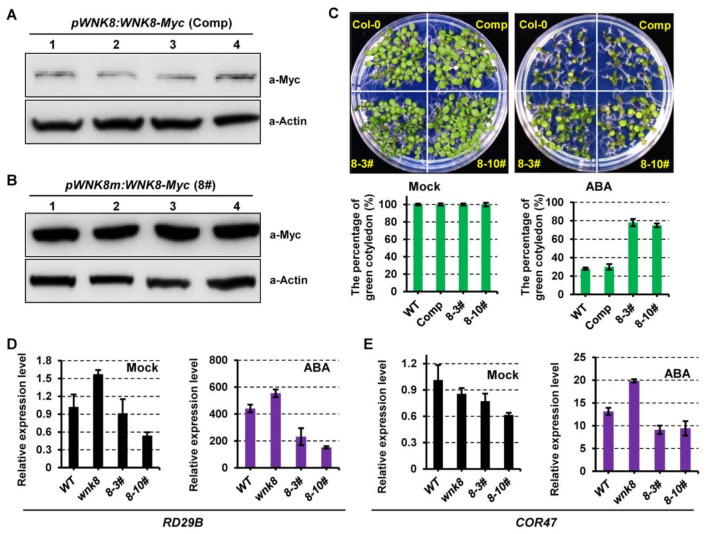
ABA sensitivity is altered under abnormal expression of WNK8. (**A**,**B**) The protein accumulation analysis of WNK8 in transgenic lines. Total proteins were extracted from the individual stable transgenic seedlings transformed with *pWNK8:WNK8* (**A**) or *pWNK8m:WNK8* (**B**), and further probed with antibodies α–Myc and α–Actin. (**C**) Photographs of seedlings grown on different media in the absence or presence of ABA at day 10 after stratification. Two independent transgenic lines transformed with *pWNK8m:WNK8* (8-3# and 8-10#) and the complementation of WNK8 in *wnk8-1* line (Comp) were analyzed. Cotyledon greening percentages were recorded after germination for 10 days. Three independent experiments were conducted, and over 50 seeds of each genotype were applied in each replicate. The standard deviation of three replicates were indicated with error bars. (**D**,**E**) The expression analysis of ABA response genes in different genotypes. The expression profiles of ABA-signaling-related genes *RD29B* (**D**) and *COR47* (**E**) were examined in one-week-old seedlings in the absence or presence of 50 μM ABA for 3 h. The transcriptional levels of ABA response genes were normalized to that of *Actin2*.

Two different genetic constructs were generated to study the role of CPuORF58 in vivo. In the first construct, WNK8 expression was controlled by a 2.3-kb region upstream of the start codon, including its 5′-UTR (*pWNK8*:*WNK8*), and in the second construct, a point mutation (G→C) was introduced in the start codon ATG of CPuORF58 (*pWNK8m*:*WNK8*). In both constructs, the Myc tag was directly attached to the mORF of *WNK8*. These constructs were used to transform *wnk8-1* mutant plants. As stated above, independent transgenic lines were generated for these constructs, after which homozygous lines were established. The WNK8 protein was rarely detected in plants transformed with *pWNK8*:*WNK8*, which was used as the complementation material hereafter (Figure 5A). This was similar to the weak expression of GUS driven, by the *WNK8* promoter. In contrast, strong WNK8 expression was detected in plants transformed with *pWNK8m*:*WNK8* (Figure 5B).

These lines were further selected to evaluate the role of WNK8 in the ABA response. As expected, the complementation of WNK8 in *wnk8-1* (Comp) showed a phenotype similar to that of the wild-type phenotype. However, plants transformed with *pWNK8m*:*WNK8* exhibited much lower sensitivity to ABA (Figure 5C). We further investigated the expression patterns of the ABA response genes *RD29B* and *COR47* in these genetic backgrounds. Under normal conditions, the expression levels of the two ABA response genes were similar in all selected plants, but slightly lower in plants transformed with *pWNK8m*:*WNK8* (Figure 5D,E). After ABA treatment, the transcription levels of both *RD29B* and *COR47* were significantly increased, with the highest in the *wnk8* mutant. The expression of the two ABA response genes was also induced in plants transformed with *pWNK8m*:*WNK8* but was lower than their expression in Col-0 and *wnk8* mutant plants. This suggested that WNK8 negatively affected the expression of ABA response genes, which is consistent with a previous study [28].

### 2.6. WNK8 and RACK1 Coordinate ABA Signaling

**Figure 6:**
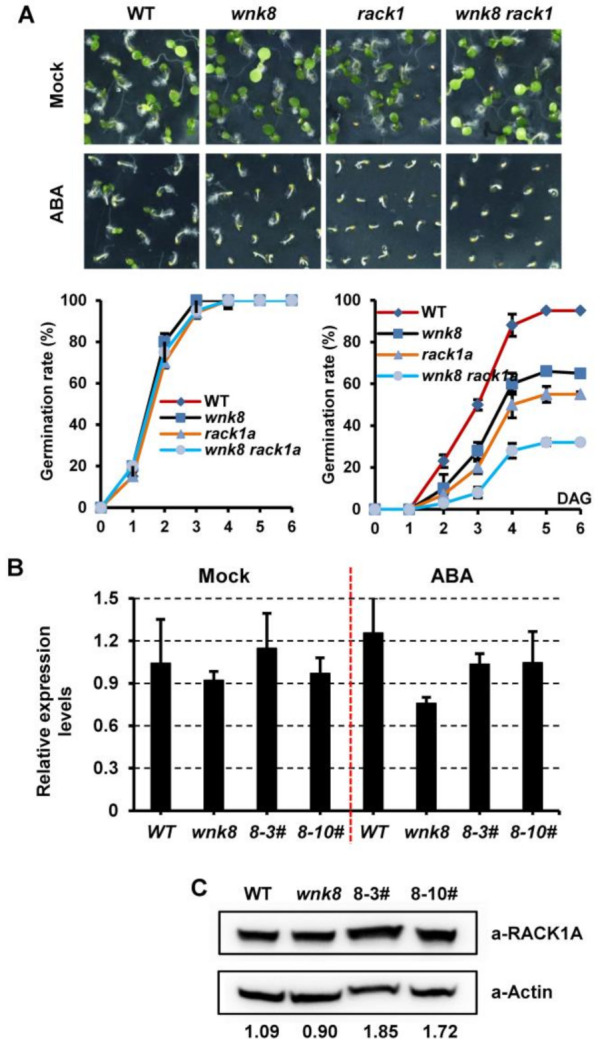
WNK8 and RACK1A coordinate ABA signaling. (**A**) WNK8 genetically interacts with RACK1A in response to ABA. Photographs of cotyledon expansion for indicated genotypes were shown in the absence or presence of 1.0 μM ABA. Time course of seed germination were recorded at the lower panel. The expression of RACK1A in WNK8 related genetic background was examined at the transcriptional (**B**) and translational levels (**C**).

Receptor for activated C kinase1 (RACK1) has been suggested to act downstream of WNK8 to regulate flowering and glucose responsiveness as well as to play a negative role in ABA signaling [17,35]. Hence, we examined whether WNK8 and RACK1 function together in coordinating ABA signaling. Seed germination analysis showed that both *wnk8-1* and *rack1a-2* were more sensitive to ABA than Col-0 plants (Figure 6A). This was different from the opposing flowering and glucose phenotypes of *rack1a-2* and *wnk8-1* single mutants [17]. Interestingly, the double mutant *rack1a-2 wnk8-1* was even more sensitive to ABA in seed germination than the single mutant. This suggests that WNK8 and RACK1 function in the same pathway to regulate the ABA response.

WNK8-mediated phosphorylation negatively affects the stability of the RACK1 protein [17]. Thus, we examined the expression of *RACK1* in different genetic backgrounds related to WNK8. The results showed that transcription of *RACK1A* was constant in Col-0, *wnk8-1* mutant, and plants transformed with *pWNK8m*:*WNK8* (Figure 6B). The protein levels of RACK1A were also similar in *Col-0* and *wnk8-1* mutant plants (Figure 6C). However, the accumulation of RACK1A was higher in plants with high WNK8 expression. This indicated the occurrence of other kinds of posttranslational modifications that may contribute to the stability of the RACK1A protein and counteract the effect of WNK8-mediated phosphorylation.

## 3. Discussion

Plants have developed diverse environmental adaptation strategies for their development and survival. In general, they adapt to alterations in environmental conditions by controlling their gene expression profiles through both transcriptional regulation and post-transcriptional regulation. It is widely known that different regulatory elements are present in the 5′-UTR of mRNAs. uORFs appear in the 5′-UTR as translational control elements, which generally attenuate the translation of the downstream mORF in most cases [36,37,38,39]. uORFs have been found in approximately 20% to 50% of eukaryotic transcripts. Approximately 50% of protein-encoding genes possess one or more uORFs in humans [39]. In plants, 24–30% of the 5′-UTR of mRNAs contains uORFs; however, only a few uORFs have been identified and characterized [40,41]. Therefore, it is necessary to further explore uORFs in plants and investigate their translational control mechanisms, which could provide a deeper understanding of plant development and adaptations to changing environments.

uORFs have been identified in many transcripts responsible for plant development, including transcription factors (TFs) and RNA processing factors [42]. In particular, genes encoding TFs are over-represented. Several studies have demonstrated that uORFs play important roles in the translational regulation of many pathways in plants including metabolic, plant morphogenesis, disease resistance, and nutrient absorption pathways [43,44,45,46,47]. For instance, several enzymes, such as S-adenosylmethionine decarboxylase (AdoMetDC) and flavin-containing polyamine oxidases (PAO), are controlled by uORFs in a small metabolite-dependent manner, which are important for the biosynthesis of polyamine (PA) and phosphocholine (PCho), respectively [48,49,50,51,52]. Some proteins are tightly regulated by uORFs, though other signals including light and plant hormones. Both *Arabidopsis* TFs AtHB1 (HOMEOBOX 1) and PHYTOCHROME-INTERACTING FACTOR 3 (AtPIF3) are light-dependent and regulated by uORFs [53,54,55]. One uORF in the 5′-UTR of the Brassinosteroid receptor protein AtBRI1 (Brassinosteroid insensitive 1) has been to be essential for the stability of BR (Brassinosteroid) levels in vivo [56]. Here, we demonstrated that CPuORF58, a highly conserved genetic element in the 5′-UTR of WNK8, is important for ABA-signaling response in plants. Our results showed that the protein level of WNK8 is closely related to the ABA response in *Arabidopsis*. The two T-DNA insertion lines *wnk8-1* and *wnk8-2*, which lacked *WNK8* transcripts, were more ABA-sensitive (Figure 1). In contrast, high expressed WNK8 expression conferred significantly less ABA sensitivity when CPuORF58 was mutated in plants (Figure 5). Thus, WNK8 expression must be tightly regulated to fulfill the demands of ABA signaling in plants.

Although uORFs are distributed in a considerable number of mRNAs, only a small number of these regulatory elements are conserved between species. Here, we showed that in all retrieved DNA sequences encoding *WNK8* homologs from both monocot and dicot species that contain known 5′-UTRs, and CPuORF58 was predicted (Figure 2B). Although the conservation of the total amino acids is not very high due to the unusual longer size (ranging from 55 aa–84 aa), it is important to note that higher conservation in some specific amino acids is species–specific (Figure 3C–E). Clear coverage peaks upstream of the uORF stop codon for the 5′-UTR of WNK8 were observed with ribosome footprinting analyses, indicating ribosome stalling in this region (Figure 2A). Here, we demonstrated the role of CPuORF58 in translational repression in vivo, which is most likely due to ribosome stalling. With frameshift mutations, the repressive activity of CPuORF58 was lost in plants, although little change was observed in the RNA sequence (Figure 3B–D). In addition, another uORF (uORF1) was identified in the 5′-UTR of *WNK8* overlapping with CPuORF58 (Figure 4A). The transient expression assay showed that either of the two uORFs was effective in triggering the translation repression of the downstream mORF, while the extent of the reduction was stronger with CPuORF58. As uORF1 partially overlapped with CPuORF58, the translated uORF1 prevented the translation of CPuORF58. Thus, the extent of suppression of reporter expression was not enhanced when both uORFs were mutated, but when they acted as mimics of the mutated uORF1 (Figure 4B–D). This indicates that at least two post-transcriptional regulatory mechanisms contribute to tight control of the WNK8 accumulation in vivo. 

RACK1A has been suggested to act downstream of WNK8 and play a negative role in ABA responses [17,35]. Here, we found that the double mutant *rack1a-2 wnk8-1* was more sensitive to ABA than either of the single mutants (Figure 6A). This indicated that WNK8 and RACK1A coordinate the ABA-signaling pathway differently from the glucose response pathway and flowering. The enhanced ABA activity in the *wnk8* mutant may lead to the repression of the RACK1 expression of, as ABA treatment resulted in rapid down-regulation of *RACK1* [35]. However, no significant change in the transcription of *RACK1A* was detected in either the control or *wnk8* mutant plants (Figure 6B). A similar expression of *RACK1A* was also found in plants with high WNK8 expression. It has been suggested that WNK8 affects the protein stability of RACK1 by phosphorylation modification [17]. However, no difference in RACK1A protein abundance was observed in either *wnk8* mutant or the wild-type plants, which is consistent with the results of a previous study. Instead, more RACK1A proteins were detected under high WNK8 expression. It is possible that the dominant effect of certain modifications contributes to stable RACK1A expression, even under high WNK8 expression. ABA represses *RACK1* at the transcriptional level, but also enhances the sumoylation of RACK1, which increases the stability of RACK1 in vivo [57]. As the ABA core receptor, PYR1 could be phosphorylated by WNK8 at several residues close to the ABA-binding site and may affect the ABA-affinity, leading to more ABA accumulation in cells [28]. It has also been suggested that WNK proteins are helpful in maintaining ABA homeostasis. GmWNK1 interacts with the putative ABA 8′-hydroxylase protein GmCYP707A1, and enhanced levels of endogenous ABA were observed in transgenic lines [22]. We suspected an accumulation of ABA when WNK8 proteins were highly expressed, therefore enhancing the protein stability of RACK1A. It remains to be investigated whether RACK1 sumoylation increases with increase in WNK8 expression. Moreover, it would be interesting to examine the ABA levels in these genetic lines in future studies.

## 4. Materials and Methods

### 4.1. Plant Materials and Growth Conditions

All plants used for transformation in this study are with the *Arabidopsis* ecotype Columbia-0 (Col-0). The mutants *wnk8-1* (SALK_206987C) and *wnk8-2* (SALK_103318C) in the Col-0 ecotype background, were supported by the Arabidopsis Biological Resource Center (http://www.arabidopsis.org accessed on 16 August 2021). The mutant *rack1a-2* (SALK_073786C) was the gift from Jiafu Jiang in Nanjing Agricultural University. Seeds were surfaced sterilized and grown in Murashige and Skoog (MS) medium with 1% sucrose and 0.8% phyto agar. Plates were kept for 3 days at 4 °C in the dark before moved to a culture room with 22 °C under a long-day (16/8 h light/dark) photoperiod. Seven-day-old seedlings were grown in the culture room with same conditions as previously stated. The *wnk8-1* was crossed with *rack1a-2* for generation of the double mutant *wnk8-1 rack1a-2*.

For the seed germination assay, seeds were surfaced sterilized and placed on the half strength MS medium in the absence or presence of various concentrations of ABA, and transferred to the culture room. The seed germination was calculated after stratification, and cotyledon expansion and greening were scored at the indicated time intervals.

### 4.2. RNA/DNA Extraction and Analysis

For genotyping of the T-DNA insertion mutants, leaves from two-week-old seedlings were harvested for DNA extraction. Edwards buffer (20 mM Tris-HCl (pH 7.5), 250 mM NaCl, 25 mM EDTA, and 0.5% SDS) was applied for quick one-step DNA extraction following the protocol. The genotyping PCR using gene-specific primers and a T-DNA primer LBa1 for mutant lines were performed using PCR mix (Vazyme, Nanjing, China). The insertion site was confirmed by DNA sequencing.

For quantitative reverse transcription-PCR (qRT-PCR), the total RNA was extracted from the *Arabidopsis* seedlings or different organs and tissues using ReliaPrep™ RNA Miniprep System (Promega, Madison, WI, USA). For cDNA synthesis, the first-strand cDNA was synthesized using 2 μg total RNA with NovoScript^®^Plus All-in-one 1st Strand cDNA Synthesis SuperMix (Novoprotein, Shanghai, China) following the instructions. The qRT-PCR was carried out with SYBR Green Master Mix (YEASEN, Shanghai, China) on Step-one Plus^TM^ (Applied Biosystems, Carlsbad, CA, USA). The 2^−ΔΔCT^ method was applied to quantify the gene expression levels [58]. *Actin2* was used as an internal control. All primers used are listed in Table S1.

### 4.3. Plasmids Construction and Plant Transformation

#### 4.3.1. *pWNK8:GUS* and *pWNK8m:GUS*

The native version of the *WNK8* promoter (2.3 kb upstream of WNK8 translation start site) was amplified and the mutated version was assembled by overlapping PCR amplification using specific oligonucleotides (Supplemental Table S1). The PCR products were cloned into the *Hind*III and *Nco*I sites of *pCAMBIA1301*, replacing the 35S CaMV. The correct insertion was verified by sequencing.

#### 4.3.2. *pWNK8:WNK8* and *pWNK8m:WNK8*

To generate the plasmid for the native expression of WNK8, the genomic region including 2.3 kb upstream of ATG plus introns and exons were cloned with nuclear sequence (GAGCAGAAACTCATCTCTGAAGAGGATCTG) for Myc tag (EQKLISEEDL) included in the reverse primer. The mutated version of the *WNK8* was assembled overlapping PCR. Both PCR products were cloned into pDONOR221 using Gateway™ BP Clonase™ Enzyme Mix (Thermo Scientific, Waltham, MA, USA), and further cloned into the pGWB4 plant expression vector using Gateway™ LR Clonase™ II Enzyme mix (Thermo Scientific, Waltham, MA, USA).

#### 4.3.3. *35S:Native-uORF:GFP* and *35S:FS-uORF:GFP*

The WNK8 5′-UTR was amplified, and the indicated mutations were introduced by overlapping using specific oligonucleotides. All the PCR products were introduced into pDONOR221 using Gateway™ BP Clonase™ Enzyme Mix, and further cloned into the pGWB405 to produce C-terminal GFP-tagged fusion proteins under the control of the 35S promoter using Gateway™ LR Clonase™ II Enzyme mix. All the constructs were confirmed by sequencing.

For plasmids construction, all genes were amplified using Tks Gflex™ DNA polymerase (Takara, Shiga, Japan) with corresponding primers listed in Table S1. The plant expression constructs were introduced into the *Agrobacterium tumefaciens* GV3101 strain. Plant transformation was applied with the floral dipping method [59]. Further selection of transformed plants was performed in petri dishes with MS medium supplemented with the appropriate selector chemical (50 mg L^−1^ kanamycin or 25 mg L^−1^ hygromycin). Three or four homozygous T3 and T4 independent lines for each construct were further reproduced and used to analyze the gene expression levels and phenotypes.

### 4.4. Histochemical GUS Staining and GUS Activity Measurement

GUS staining was performed with commercial kit by following the protocol (Coolaber, Beijing, China). In brief, young seedlings, mature leaves, and flowers were immersed into fresh prepared GUS staining buffer, further with vacuum for 5 min, and then plants were kept at 37°C for 24 h. Chlorophyll was removed from the plant tissues with 70% ethanol.

To examine the GUS activity, a plant-GUS ELISA kit was applied following the protocol (Abmart, Shanghai, China). The fresh plant tissue samples were grinded with 0.75% NaCl solution. After centrifuge for 10 min with 3000 rpm, the supernatant was collected and further incubated with reagents provided in kit. The OD values from the standard and test samples were measured with a wavelength of 450 nm. The GUS activity in each sample can be normalized per unit tissue weight.

### 4.5. Immunoblotting

Seedlings of stable transgenic lines expressed the corresponding GFP or Myc fusions were freeze grounded into powder and homogenized in total protein buffer (20 mmo1/L Tris-HCl (pH 7.5), 150 mmol/L NaCl, 2 mmol/L EDTA) with protease inhibitor cocktail in DMSO (YEASEN, Shanghai, China). Lysates were incubated on ice for 20 min and clarified by centrifugation at 15,000 g for 15 min at 4°C. For immunoblotting, samples were separated on 12% SDS polyacrylamide gel and transferred to PVDF membranes. The membranes were then blocked with 5% (g/v) defatted milk in TBST buffer (10 mM Tris-HCl (pH 7.4), 150 mM NaCl, 0.05% Tween 20) and probed with using appropriate antibodies including 1:6000 dilution α-GFP conjugated with HRP (MBL, Nagano, Japan), 1:5000 dilution of α-RACK1 (PhytoAB, SAN JOSE, CA, USA), α-Myc (MBL, Nagano, Japan) and α-Actin2 (Sangon, Shanghai, China) overnight at 4°C. Then the samples were washed with TBST buffer for three times and visualized using the ECL (Amersham™, Boston, MA, USA). Actin protein was used as the internal control. Image J2 was applied for quantification the intensity of signals [60].

### 4.6. Database Screening Analysis

The BLASTP program was applied for a search for *Arabidopsis* WNK proteins on the whole genome sequences from the Phytozome database (https://phytozome.jgi.doe.gov accessed on 16 August 2021), with AtWNK8 as the query sequence. Corresponding genomic DNA (gDNA) and coding DNA (cDNA) sequences of the WNK8s from the selected plant genome were retrieved. The uORF nucleotides and predicted amino acid sequences were aligned using the ClustalW software (version 2.0.12) [61]. The sequence logos were generated with MEME (https://meme-suite.org/meme/tools/meme accessed on 16 August 2021) with default setting. The GWIPS-viz (https://gwips.ucc.ie/ accessed on 16 August 2021) was applied to visualize the RNA-seq and ribosome profiling data of WNK8. The data exploration tool ATHENA (http://athena.proteomics.wzw.tum.de:5002/master_arabidopsisshiny/ accessed on 16 August 2021) was used for comparing the proteome and transcriptome data of WNK8.

## 5. Conclusions

In this study, we found that t*Arabidopsis* WNK8 is post-transcriptionally regulated by the conserved peptide (CPuORF58) located in its 5′ untranslated region (5′-UTR). Together with another uORF, CPuORF58 represses WNK8 expression to fulfill the demands of ABA response in plants. Moreover, WNK8 and its downstream target RACK1 synergistically coordinate ABA signaling rather than antagonistically modulating plant glucose response and flowering. This study provides a better understanding of the role of uORFs in plant development and adaptation to changing environments.

## Data Availability

The whole genome sequences for *WNK8* and the homologs can be found in the Phytozome database (https://phytozome.jgi.doe.gov accessed on 16 August 2021). The RNA-seq and ribosome profiling data for genes from *Arabidopsis* can be visualized in GWIPS-viz (https://gwips.ucc.ie/ accessed on 16 August 2021). And proteome and transcriptome for genes from *Arabidopsis* can be obtained from ATHENA (http://athena.proteomics.wzw.tum.de:5002/master_arabidopsisshiny/ accessed on 16 August 2021).

## Supplementary Materials

The following are available online at https://www.mdpi.com/article/10.3390/ijms221910683/s1.

## References

[1] Xu B.E., English J.M., Wilsbacher J.L., Stippec S., Goldsmith E.J., Cobb M.H. (2000). WNK1, a novel mammalian serine/threonine protein kinase lacking the catalytic lysine in subdomain II. J. Biol. Chem..

[2] Verissimo F., Jordan P. (2001). WNK kinases, a novel protein kinase subfamily in multi-cellular organisms. Oncogene.

[3] Gamba G. (2005). Role of WNK kinases in regulating tubular salt and potassium transport and in the development of hypertension. Am. J. Physiol. Renal Physiol..

[4] Wilson F.H. (2001). Human hypertension caused by mutations in WNK kinases. Science.

[5] McCormick J.A., Ellison D.H. (2011). The WNKs: Atypical protein kinases with pleiotropic actions. Physiol. Rev..

[6] Huang C.L., Cheng C.J. (2015). A unifying mechanism for WNK kinase regulation of sodium-chloride cotransporter. Pfluger. Arch. Pflug. Arch. Eur. J. Phy..

[7] Richardson C., Rafiqi F.H., Karlsson H.K., Moleleki N., Vandewalle A., Campbell D.G., Morrice N.A., Alessi D.R. (2008). Activation of the thiazide-sensitive Na^+^-Cl^−^ cotransporter by the WNK-regulated kinases SPAK and OSR1. J. Cell. Sci..

[8] Yang C.L., Angell J., Mitchell R., Ellison D.H. (2003). WNK kinases regulate thiazide-sensitive Na-Cl cotransport. J. Clin. Investig..

[9] Rinehart J., Kahle K.T., de Los Heros P., Vazquez N., Meade P., Wilson F.H., Hebert S.C., Gimenez I., Gamba G., Lifton R.P. (2005). WNK3 kinase is a positive regulator of NKCC2 and NCC, renal cation-Cl- cotransporters required for normal blood pressure homeostasis. Proc. Natl. Acad. Sci. USA.

[10] Sie Z.L., Li R.Y., Sampurna B.P., Hsu P.J., Liu S.C., Wang H.D., Huang C.L., Yuh C.H. (2020). WNK1 kinase stimulates angiogenesis to promote tumor growth and metastasis. Cancers.

[11] Lai J.G., Tsai S.M., Tu H.C., Chen W.C., Kou F.J., Lu J.W., Wang H.D., Huang C.L., Yuh C.H. (2014). Zebrafish WNK lysine deficient protein kinase 1 (wnk1) affects angiogenesis associated with VEGF signaling. PLoS ONE.

[12] Tang B.L. (2016). (WNK) ing at death: With-no-lysine (Wnk) kinases in neuropathies and neuronal survival. Brain. Res. Bull..

[13] Wang Y., Liu K., Liao H., Zhuang C., Ma H., Yan X. (2008). The plant WNK gene family and regulation of flowering time in *Arabidopsis*. Plant Biol..

[14] Manuka R., Saddhe A.A., Kumar K. (2015). Genome-wide identification and expression analysis of WNK kinase gene family in rice. Comp. Biol. Chem..

[15] Wang Y., Suo H., Zheng Y., Liu K., Zhuang C., Kahle K.T., Ma H., Yan X. (2010). The soybean root-specific protein kinase GmWNK1 regulates stress-responsive ABA signaling on the root system architecture. Plant J..

[16] Cao S., Hao P., Shu W., Wang G., Xie Z., Gu C., Zhang S. (2019). Phylogenetic and expression analyses of With-No-Lysine kinase genes reveal novel gene family diversity in fruit trees. Hortic. Plant J..

[17] Urano D., Czarnecki O., Wang X., Jones A.M., Chen J.G. (2015). *Arabidopsis* receptor of activated C kinase1 phosphorylation by WITH NO LYSINE8 KINASE. Plant Physiol..

[18] Nakamichi N., Murakami-Kojima M., Sato E., Kishi Y., Yamashino T., Mizuno T. (2002). Compilation and characterization of a novel WNK family of protein kinases in *Arabidopsis thaliana* with reference to circadian rhythms. Biosci. Biotechnol. Biochem..

[19] Murakami-Kojima M., Nakamichi N., Yamashino T., Mizuno T. (2002). The APRR3 component of the clock-associated APRR1/TOC1 quintet is phosphorylated by a novel protein kinase belonging to the WNK family, the gene for which is also transcribed rhythmically in *Arabidopsis thaliana*. Plant Cell Physiol..

[20] Kumar K., Rao K.P., Biswas D.K., Sinha A.K. (2011). Rice WNK1 is regulated by abiotic stress and involved in internal circadian rhythm. Plant Signal. Behav..

[21] Hong-Hermesdorf A., Brüx A., Grüber A., Grüber G., Schumacher K. (2006). A WNK kinase binds and phosphorylates V-ATPase subunit C. Febs. Lett..

[22] Wang Y., Suo H., Zhuang C., Ma H., Yan X. (2011). Overexpression of the soybean GmWNK1 altered the sensitivity to salt and osmotic stress in *Arabidopsis*. J. Plant. Physiol..

[23] Zhang B., Liu K., Zheng Y., Wang Y., Wang J., Liao H. (2013). Disruption of AtWNK8 enhances tolerance of *Arabidopsis* to salt and osmotic stresses via modulating proline content and activities of catalase and peroxidase. Int. J. Mol. Sci..

[24] Xie M., Wu D., Duan G., Wang L., He R., Li X., Tang D., Zhao X., Liu X. (2014). AtWNK9 is regulated by ABA and dehydration and is involved in drought tolerance in *Arabidopsis*. Plant Physiol. Biochem..

[25] Manuka R., Karle S.B., Kumar K. (2019). OsWNK9 mitigates salt and drought stress effects through induced antioxidant systems in *Arabidopsis*. Plant Physiol. Rep..

[26] Manuka R., Saddhe A.A., Srivastava A.K., Kumar K., Penna S. (2021). Overexpression of rice OsWNK9 promotes arsenite tolerance in transgenic *Arabidopsis* plants. J. Biotechnol..

[27] Dunker F., Trutzenberg A., Rothenpieler J.S., Kuhn S., Pröls R., Schreiber T., Tissier A., Kemen A., Kemen E., Hückelhoven R. (2020). Oomycete small RNAs bind to the plant RNA-induced silencing complex for virulence. Elife.

[28] Waadt R., Jawurek E., Hashimoto K., Li Y., Scholz M., Krebs M., Czap G., Hong-Hermesdorf A., Hippler M., Grill E. (2019). Modulation of ABA responses by the protein kinase WNK8. Febs Lett..

[29] Mergner J., Frejno M., List M., Papacek M., Chen X., Chaudhary A., Samaras P., Richter S., Shikata H., Messerer M. (2020). Mass-spectrometry-based draft of the *Arabidopsis* proteome. Nature.

[30] Hayden C.A., Jorgensen R.A. (2007). Identification of novel conserved peptide uORF homology groups in *Arabidopsis* and rice reveals ancient eukaryotic origin of select groups and preferential association with transcription factor-encoding genes. BMC Biol..

[31] Ingolia N.T., Ghaemmaghami S., Newman J.R., Weissman J.S. (2009). Genome-wide analysis in vivo of translation with nucleotide resolution using ribosome profiling. Science.

[32] Michel A.M., Fox G.M., Kiran A., De Bo C., O’Connor P.B., Heaphy S.M., Mullan J.P., Donohue C.A., Higgins D.G., Baranov P.V. (2014). GWIPS-viz: Development of a ribo-seq genome browser. Nucleic Acids Res..

[33] Hinnebusch A.G., Ivanov I.P., Sonenberg N. (2016). Translational control by 5′-untranslated regions of eukaryotic mRNAs. Science.

[34] Hayashi N., Sasaki S., Takahashi H., Yamashita Y., Naito S., Onouchi H. (2017). Identification of *Arabidopsis thaliana* upstream open reading frames encoding peptide sequences that cause ribosomal arrest. Nucleic Acids Res..

[35] Guo J., Wang J., Xi L., Huang W.D., Liang J., Chen J.G. (2009). RACK1 is a negative regulator of ABA responses in *Arabidopsis*. J. Exp. Bot..

[36] Morris D.R., Geballe A.P. (2020). Upstream open reading frames as regulators of mRNA translation. Mol. Cell. Biol..

[37] Calvo S.E., Pagliarini D.J., Mootha V.K. (2009). Upstream open reading frames cause widespread reduction of protein expression and are polymorphic among humans. Proc. Natl. Acad. Sci. USA.

[38] Jeon S., Kim J. (2010). Upstream open reading frames regulate the cell cycle-dependent expression of the RNA helicase Rok1 in *Saccharomyces cerevisiae*. FEBS Lett..

[39] Kwon H.S., Lee D.K., Lee J.J., Edenberg H.J., Ahn Y.H., Hur M.W. (2001). Posttranscriptional regulation of human ADH5/FDH and Myf6 gene expression by upstream AUG codons. Arch. Biochem. Biophys..

[40] Shashikanth M., Krishna A.R., Ramya G., Devi G., Ulaganathan K. (2008). Genome-wide comparative analysis of *Oryza sativa (japonica)* and *Arabidopsis thaliana* 5′-UTR sequences for translational regulatory signals. Plant Biotech..

[41] Niu R., Zhou Y., Zhang Y., Mou R., Tang Z., Wang Z., Zhou G., Guo S., Yuan M., Xu G. (2020). uORFlight: A vehicle toward uORF-mediated translational regulation mechanisms in eukaryotes. Database.

[42] Kalyna M., Simpson C.G., Syed N.H., Lewandowska D., Marquez Y., Kusenda B., Marshall J., Fuller J., Cardle L., McNicol J. (2012). Alternative splicing and nonsense-mediated decay modulate expression of important regulatory genes in *Arabidopsis*. Nucleic Acids Res..

[43] Rosado A., Li R., van de Ven W., Hsu E., Raikhel N.V. (2012). *Arabidopsis* ribosomal proteins control developmental programs through translational regulation of auxin response factors. Proc. Natl. Acad. Sci. USA.

[44] Yang S.Y., Lu W.C., Ko S.S., Sun C.M., Hung J.C., Chiou T.J. (2020). Upstream open reading frame and phosphate-regulated expression of rice OsNLA1 controls phosphate transport and reproduction. Plant Physiol..

[45] Xu G., Yuan M., Ai C., Liu L., Zhuang E., Karapetyan S., Wang S., Dong X. (2017). uORF-mediated translation allows engineered plant disease resistance without fitness costs. Nature.

[46] Tanaka M., Takano J., Chiba Y., Lombardo F., Ogasawara Y., Onouchi H., Naito S., Fujiwara T. (2011). Boron-dependent degradation of NIP5;1 mRNA for acclimation to excess boron conditions in *Arabidopsis*. Plant Cell.

[47] Pajerowska-Mukhtar K.M., Wang W., Tada Y., Oka N., Tucker C.L., Fonseca J.P., Dong X. (2012). The HSF-like transcription factor TBF1 is a major molecular switch for plant growth-to-defense transition. Curr. Biol..

[48] Hanfrey C., Elliott K.A., Franceschetti M., Mayer M.J., Illingworth C., Michael A.J. (2005). A dual upstream open reading frame-based autoregulatory circuit controlling polyamine-responsive translation. J. Biol. Chem..

[49] Kamada-Nobusada T., Hayashi M., Fukazawa M., Sakakibara H., Nishimura M. (2008). A putative peroxisomal polyamine oxidase, AtPAO4, is involved in polyamine catabolism in *Arabidopsis thaliana*. Plant Cell Physiol..

[50] Guerrero-González M.L., Rodríguez-Kessler M., Jiménez-Bremont J.F. (2014). uORF, a regulatory mechanism of the *Arabidopsis* polyamine oxidase 2. Mol. Biol. Rep..

[51] Cruz-Ramírez A., López-Bucio J., Ramírez-Pimentel G., Zurita-Silva A., Sánchez-Calderon L., Ramírez-Chávez E., González-Ortega E., Herrera-Estrella L. (2004). The xipotl mutant of *Arabidopsis* reveals a critical role for phospholipid metabolism in root system development and epidermal cell integrity. Plant Cell.

[52] Alatorre-Cobos F., Cruz-Ramirez A., Hayden C.A., Pérez-Torres C.A., Chauvin A.L., Ibarra-Laclette E., Alva-Cortés E., Jorgensen R.A., Herrera-Estrella L. (2012). Translational regulation of *Arabidopsis* XIPOTL1 is modulated by phosphocholine levels via the phylogenetically conserved upstream open reading frame 30. J. Exp. Bot..

[53] Ribone P.A., Capella M., Arce A.L., Chan R.L. (2017). A uORF represses the transcription factor AtHB1 in aerial tissues to avoid a deleterious phenotype. Plant Physiol..

[54] Capella M., Ribone P.A., Arce A.L., Chan R.L. (2015). *Arabidopsis thaliana* HomeoBox 1 (AtHB1), a Homedomain-Leucine Zipper I (HD-Zip I) transcription factor, is regulated by PHYTOCHROME-INTERACTING FACTOR 1 to promote hypocotyl elongation. New Phytol..

[55] Dong J., Chen H., Deng X.W., Irish V.F., Wei N. (2020). Phytochrome B induces intron retention and translational inhibition of PHYTOCHROME-INTERACTING FACTOR3. Plant Physiol..

[56] Zhang H., Si X., Ji X., Fan R., Liu J., Chen K., Wang D., Gao C. (2018). Genome editing of upstream open reading frames enables translational control in plants. Nat. Biotechnol..

[57] Guo R., Sun W. (2017). Sumoylation stabilizes RACK1B and enhance its interaction with RAP2.6 in the abscisic acid response. Sci. Rep..

[58] Pfaffl M.W. (2001). A new mathematical model for relative quantification in real-time RT-PCR. Nucleic Acids Res..

[59] Clough S.J., Bent A.F. (1998). Floral dip: A simplified method for Agrobacterium-mediated transformation of *Arabidopsis thaliana*. Plant J..

[60] Rueden C.T., Schindelin J., Hiner M.C., DeZonia B.E., Walter A.E., Arena E.T., Eliceiri K.W. (2017). ImageJ2: ImageJ for the next generation of scientific image data. BMC Bioinfor..

[61] Larkin M.A., Blackshields G., Brown N.P., Chenna R., McGettigan P.A., McWilliam H., Valentin F., Wallace I.M., Wilm A., Lopez R. (2007). Clustal W and Clustal X version 2.0. Bioinformatics.

