# Supplementary figures and images for: Upstream Open Reading Frame Mediated Translation of WNK8 Is Required for ABA Response in Arabidopsis
Source: Int J Mol Sci. 2021 Oct 1;22(19):10683. doi: 10.3390/ijms221910683 (PMC8509022; doi:10.3390/ijms221910683)

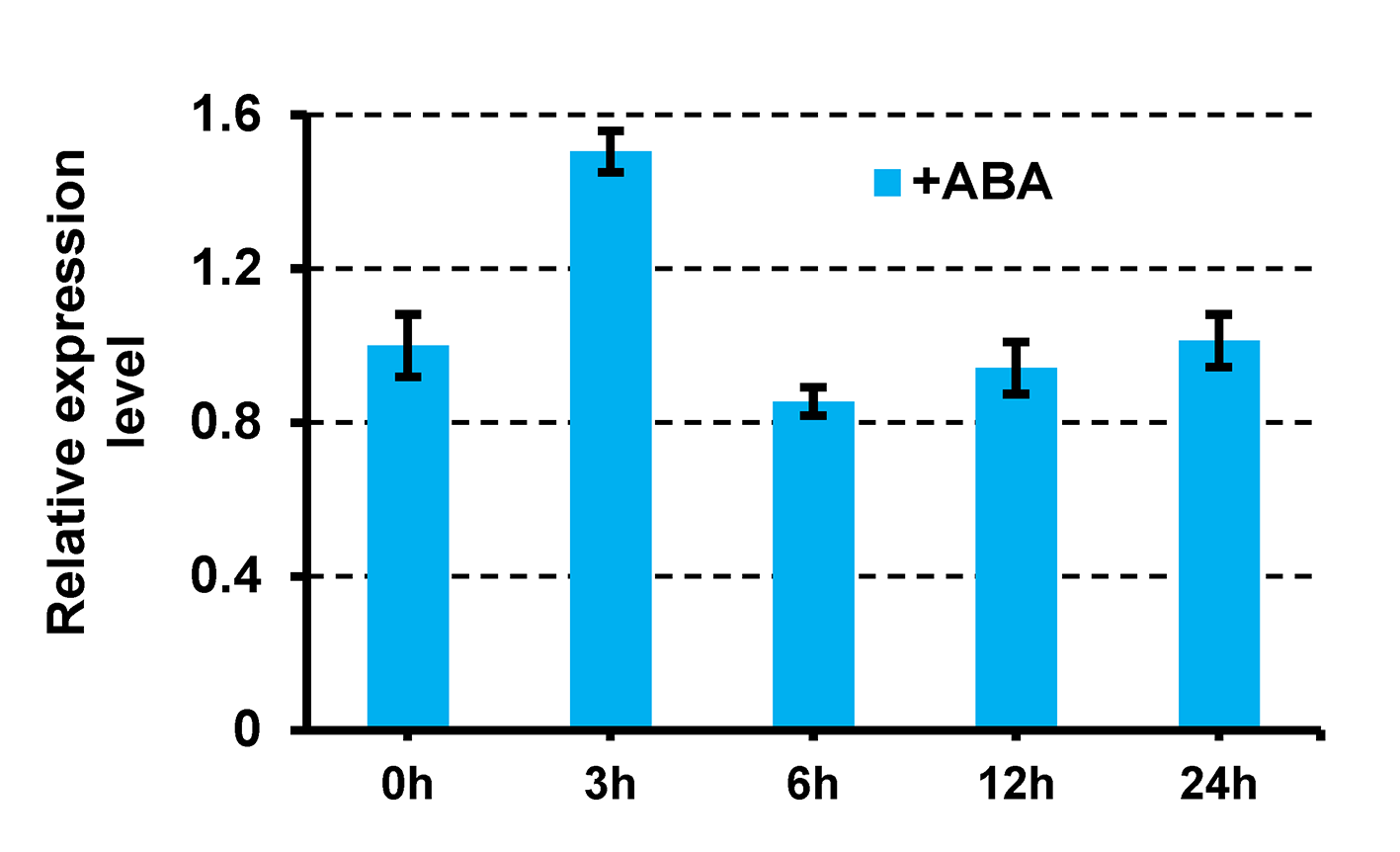

Supplement: Supplementary file 1 [file ijms-22-10683-s001.zip › Supplementary Figure S1.tif]

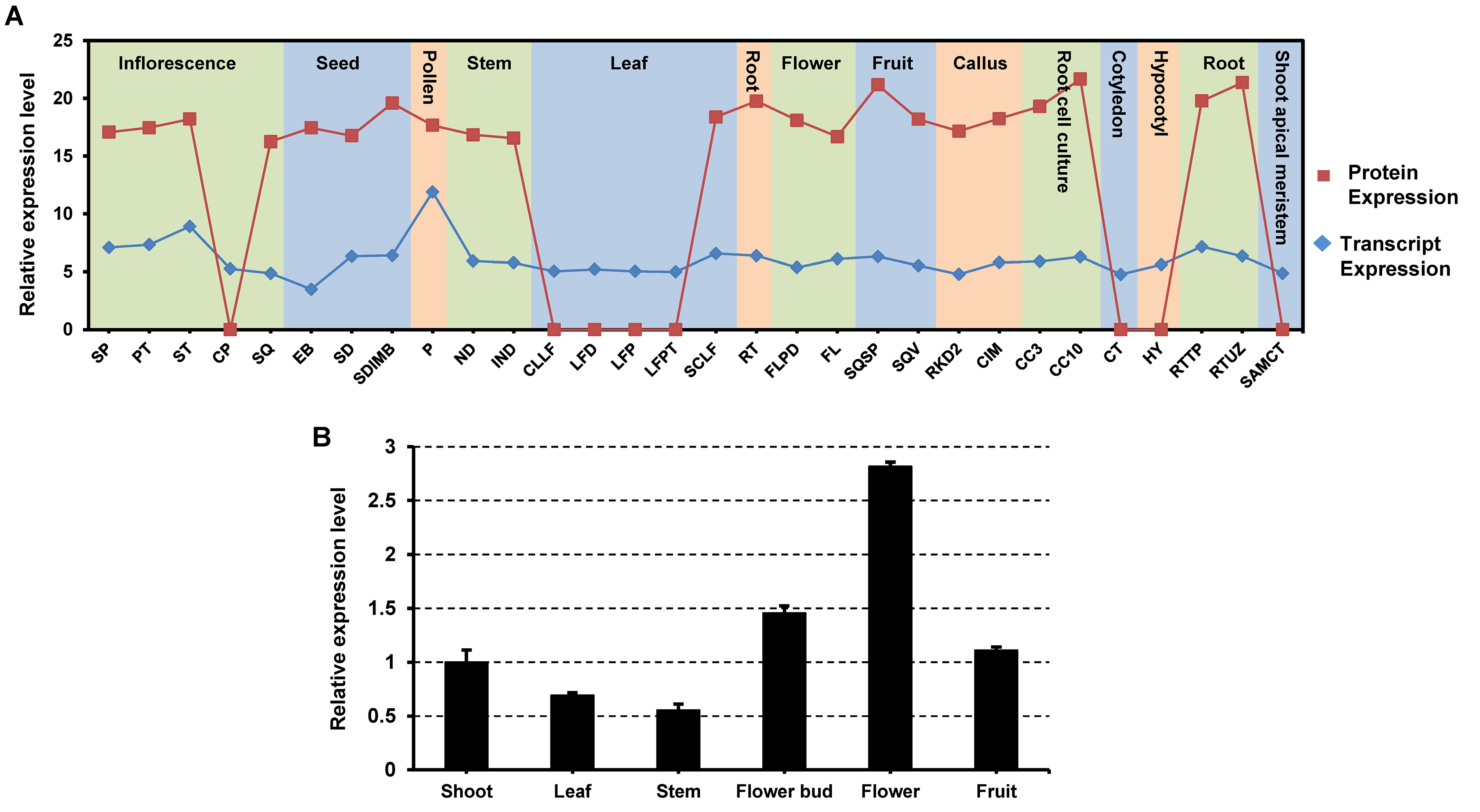

Supplement: Supplementary file 1 [file ijms-22-10683-s001.zip › Supplementary Figure S2.tif]

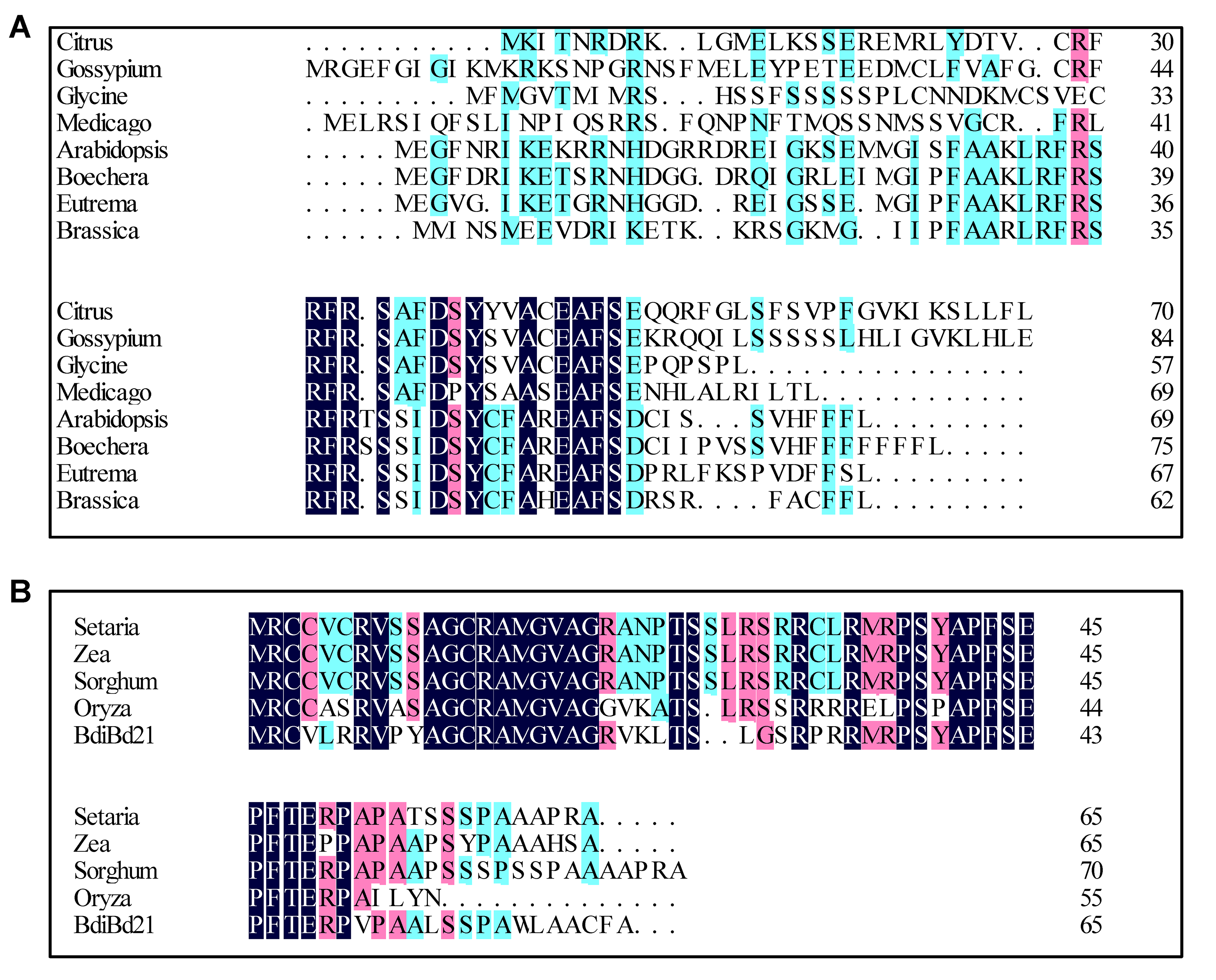

Supplement: Supplementary file 1 [file ijms-22-10683-s001.zip › Supplementary Figure S3.tif]

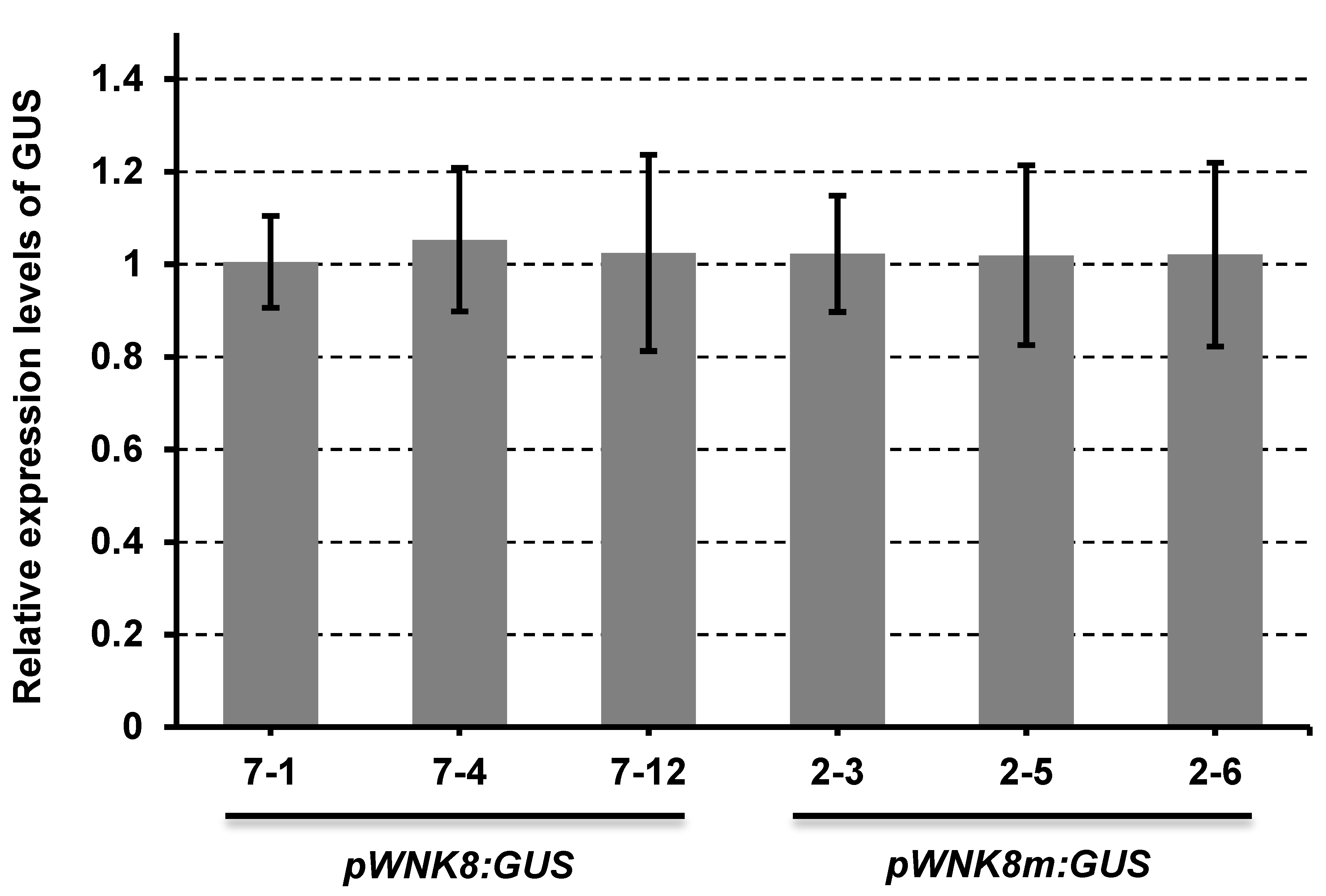

Supplement: Supplementary file 1 [file ijms-22-10683-s001.zip › Supplementary Figure S4.tif]
